# Genetic diversity of *Listeria monocytogenes* isolates in Colombia from 2010 until 2017 and comparison against the PulseNet regional surveillance network

**DOI:** 10.7705/biomedica.7863

**Published:** 2026-04-28

**Authors:** Paula Díaz-Guevara, Julieth Carolina Gamba, Diego Prada, Lucy Angeline Montaño, Edna Catering Rodriguez, Efrain Montilla-Escudero, Paloma Cuenca, Sandra Saavedra, Nathaly Rozo, Monica Prieto, Claudia Martinez, Isabel Chinen

**Affiliations:** 1 Grupo de Microbiología, Instituto Nacional de Salud, Bogotá, D.C., Colombia Instituto Nacional de Salud Grupo de Microbiología Instituto Nacional de Salud Bogotá, D.C. Colombia; 2 Grupo de Mortalidad Perinatal y Neonatal Tardía, Equipo de Maternidad Segura, Subdirección de Prevención y Vigilancia y Control en Salud Pública, Instituto Nacional de Salud, Bogotá, D.C., Colombia Instituto Nacional de Salud Grupo de Mortalidad Perinatal y Neonatal Tardía, Equipo de Maternidad Segura Subdirección de Prevención y Vigilancia y Control en Salud Pública Instituto Nacional de Salud Bogotá, D.C. Colombia; 3 PulseNet Latin America and Caribbean Network, Subregional, Instituto Carlos Malbrán, Buenos Aires, Argentina Instituto Carlos Malbrán Instituto Carlos Malbrán Buenos Aires Argentina

**Keywords:** Listeria monocytogenes, listeriosis, electrophoresis, gel, pulsed field, whole genome sequencing, drug resistance, perinatal mortality, meningitis, foodborne diseases., Listeria monocytogenes, listeriosis, electroforesis en gel de campo pulsado, secuenciación completa del genoma, resistencia a medicamentos, mortalidad perinatal, meningitis, enfermedades transmitidas por los alimentos.

## Abstract

**Introduction.:**

Listeriosis is a food-borne disease associated with *Listeria monocytogenes*, with high mortality in individuals with weakened immune systems. In Colombia, notification of the disease is not mandatory.

**Objective.:**

To characterize phenotypically and genomically the clinical isolates of *L. monocytogenes* isolated from 2010 to 2017 in Colombia.

**Materials and methods.:**

A total of 63 of the 443 isolates of *L. monocytogenes* were analyzed by laboratory surveillance of the *Grupo de Microbiología at the Instituto Nacional de Salud*. Antimicrobial susceptibility, serological test (O-H) antigens and PCR, pulsed-field gel electrophoresis (PFGE)-*Apal* enzyme as well as whole genome sequencing (WGS) were performed. Fatalities attributed to *L. monocytogenes* were identified using SIVIGILA.

**Results.:**

An estimated 50.73% of the isolates were females, in adults ≥ 15 years old (54%) and were recovered from blood cultures (58.7%), sensitive to penicillin and to trimethoprim sulfamethoxazole. Of the isolates, 77.8% were of the complex serogroup 4b-4d-4e; 42 patterns PFGE-ApaI were determined. Genomic sequencing of five isolates identified multilocus sequencing typing (MLST) ST1, ST2, ribosomal MLST (rMLST) 15992, 15983, 15959, 98481, clonal complex CC1, CC2, lineage I, and serotype 4b *in silico*. Virulence genes identified included pathogenicity island LIPI-1, LIPI-3 and internalins. Resistance genes included *fosX, fosS-2, lin, norB* and *mprF*. The plasmid J1776, and prophages vB_LmoS_293, LP_030_2, LP_030_3, A118 were identified. Global phylogenetic analysis identified that the closest sequences belonged to lineage I, and were from France, Chile, Perú and India.

**Conclusions.:**

Strengthening epidemiological and laboratory surveillance would improve the control measures in public health.

## Resumen

**Introducción.:**

La listeriosis es una enfermedad transmitida por alimentos asociada con la infección por *Listeria monocytogenes*, con gran mortalidad en individuos con el sistema inmunológico debilitado. En Colombia, no es obligatorio notificar la enfermedad.

**Objetivo.:**

Caracterizar fenotípica y genómicamente los aislamientos clínicos de *L. monocytogenes* realizados entre el 2010 y el 2017 en Colombia.

**Materiales y métodos.:**

Se analizaron 63 de 443 aislamientos de *L. monocytogenes* de la Vigilancia por Laboratorio del Grupo de Microbiología del Instituto Nacional de Salud. Se practicaron pruebas de sensibilidad antimicrobiana, serología para antígenos (O-H) y PCR, PFGE-Apal, así como secuenciación del genoma completo. Los casos fatales atribuidos a *L. monocytogenes* fueron identificados en el Sistema Nacional de Vigilancia en Salud Pública (SIVIGILA).

**Resultados.:**

De los aislamientos, la mayoría correspondió al sexo femenino (n = 32; 50,7%). En cuanto a la distribución por edad, la mayoría de los casos se presentó en el grupo de 15 a 54 años (n = 21; 33,3%). La mayoría de los aislamientos provino de hemocultivos (n = 37; 58,7%), con sensibilidad a la penicilina y al trimetoprim-sulfametoxazol.

El 77,8% de los aislamientos correspondió al complejo serogrupo 4b-4d-4e; se determinaron 42 patrones PFGE-Apal. La secuenciación genómica de cinco aislamientos permitió identificar, mediante tipificación por secuenciación multilocus (MLST), los tipos ST1 y ST2; mediante MLST ribosómico (rMLST), los tipos 15992,15983,15959 y 98481; los complejos clónales CC1 y CC2; el linaje I y el serotipo 4b *in silico*. Los genes de virulencia identificados incluyeron islas de pathogencidad LIPI-1, LIPI-3 e internalinas; los genes de resistencia fueron *fosX. fosS-2*, Un, *norB* y *mprF*. Se identificaron el plásmido J1776 y los profagos vB_LmoS_293, LP_030_2, LP_030_3 y Al 18. El análisis filogenético global mostró que las secuencias más cercanas pertenecen al linaje I y provienen de Francia, Chile, Perú e India.

**Conclusión.:**

El fortalecimiento en el seguimiento epidemiológico y la tipificación permiten mejorar las medidas de control en salud pública.

*Listeria monocytogenes* is an opportunistic food-borne bacterial pathogen and the etiologic agent of listeriosis disease. Listeriosis is an opportunistic invasive illness which occurs in immunocompromised individuals, elderly persons, newborns, and pregnant women. A noninvasive form of listeriosis is known to occur in immunocompetent individuals with febrile gastroenteritis and flu-like symptoms ^1^^-^^4^.

In 2010, it was reported that the annual incidence of listeriosis varies from 0.337 per 100 000 people, depending on the countries and regions of the world ^4^^,^^5^. in 2023, in the United States it was about 0.31 cases per 100,000, and for the European Union member states and European Economic Area countries the notification rate was 0.66 per 100 000 with a 20-30% case mortality rate in high risk groups ^1^^,^^6^.

Acquisition of the disease is principally due to the consumption of contaminated foods -meat, vegetables, ready-to-eat products, seafood, pasteurized milk, ice cream and spreadable cheese products ^1^^,^^2^, and also zoonotic transmission ^7^. *Listeria monocytogenes* has been classified into 14 distinct serotypes (1/2a, 1/2b, 1 /2c, 3, 3a, 3b, 3c, 4a, 4ab, 4b, 4c, 4d, 4e and 7) ^8^ among these, a subset of strains belonging to serotypes 1/2a, 1/2b and 4b are particularly associated with cases of human listeriosis ^8^^-^^10^.

In foodborne cases, serogroup 1/2a is frequently observed in sporadic instances of listeriosis. However, the majority of outbreaks in human populations stem from the highly virulent serotype 4b ^9^. These outbreaks are often linked to a restricted set of epidemic clones, highlighting the significant impact of specific strain subtypes in propagating the disease ^11^.

*Listeria monocytogenes* is sensitive to most antibiotics, except for broad-spectrum cephalosporins such as cefotaxime and monobactams ^12^. Serological methods have been used for a long-term surveillance of human listeriosis ^13^, PCR has been recommended as an alternative to conventional serotyping ^8^. Pulsed-field gel electrophoresis (PFGE) is the standard subtyping method for *L. monocytogenes* due to sensitive discrimination between isolates provided by this technique ^14^^,^^15^.

Recently, whole genome sequencing (WGS) has demonstrated better resolution in epidemiological genomics-based pathogen surveillance. It defines the population structure and reveals the extent of heterogeneity in virulence and stress resistance genomic features among clinical and food isolates. WGS facilitates outbreak investigation and enhances the capacity for international circulation of strains. It also improves the identification of characteristics such as persistence and hypervirulence in isolates ^16^^-^^19^.

The clonal structure of *L. monocytogenes* is notably cohesive, comprised of four distinct phylogenetic lineages (I, II, III, IV), encompassing 13 serotypes and exhibiting numerous clonal complexes (CC) and sublineages (SL). These designations are established through multilocus sequence typing (MLST) and core genome MLST (cgMLST) ^19^.

According to the laboratory surveillance of the *Grupo de Microbiología* at the *Instituto Nacional de Salud*, from 1991 to 2017, 443 strains of *L. monocytogenes* were isolated from diverse clinical specimens or samples, blood cultures (n = 237; 53.5%), cerebrospinal fluid (n = 128; 28.9%), amniotic fluid (n = 8; 1.8%) and others (n = 70; 15.8%). This suggests the invasive capacity of the pathogen. Most of these isolates were reported in Bogotá (n = 192; 43.3%) and Valle del Cauca (n = 71; 16 %); the remainder were from other municipalities or departments (n = 180; 40.6%).

The objective of our study was to describe the epidemiological, phenotypical, and genotypic characteristics of a selected set of clinical isolates of *L. monocytogenes* in Colombia from 2010 to 2017 and describe the regional circulation through an international surveillance network.

## Materials and methods

### Human bacterial isolates

In the framework of the Programa de vigilancia por el laboratorio de bacteriología general, 443 isolates *L. monocytogenes* were received at the Grupo de Microbiología of the *Instituto Nacional de Salud* from 2010 to 2017. Out of these, 63 clinical isolates were selected for this study, based on the annual proportional distribution of the cases reported by each public health laboratory (table 1, table 2). In addition to these isolates, data routinely collected following the protocol forfoodborne diseases, demographic, clinical and microbiological information was also provided.

**Table 1.** Epidemiological characteristics from patients with clinical isolates of *Listeria monocytogenes* (N = 63)

Epidemiological characteristics	Demographic and clinical information	n	(%)
Age	Perinatal (≥ 22weeks - 7 days after delivery)	5	(7,94)
	Newborn (8 days -1 month)	14	(22,2)
	2 months - 1 year	1	(1,59)
	2-14 years	4	(6,34)
	15-54 years	21	(33,3)
	> 55 years	13	(20,63)
	No data	5	(7,94)
	Total	63	(100)
Gender	Male	28	(44,4)
	Female	32	(50,73)
	No data	3	(4,76)
	Total	63	(100)
Source sample	Blood culture	37	(58,73)
	Cerebrospinal fluid	12	(19,05)
	Amniotic fluid	4	(6,35)
	Ascitic fluid	1	(1,59)
	Stools	9	(14,28)
	Total	63	(100)

**Table 2:** Serotype and serogroup determination by multiplex PCR and serological testing of clinical isolates of *Listeria monocytogenes*, according to the identification scheme of the *Grupo de Microbiología, Instituto Nacional de Salud*

PCR serogroup complex	Serological identification of serotype	Location	n (%)	
4b,4d,4e	4b,4e	Antioquia	6	(9.52)
		Bogotá	9	(14.28)
		Boyacá	2	(3.17)
		Caquetá	1	(1.59)
		Meta	1	(1.59)
		Nariño	2	(3.17)
		Santander	2	(3.17)
		Valle	6	(9.52
	Subtotal		29	(46)
	4d,4e	Antioquia	4	(6.35)
		Bogotá	2	(3.17)
		Bolívar	1	(1.59)
		Boyacá	2	(3.17)
		Caldas	1	(1.59)
		Caquetá	1	(1.59)
		Nariño	3	(4.76)
		Risaralda	1	(1.59)
		Santander	2	(3.17)
		Valle	2	(3.17)
	Subtotal		19	(30.2)
	4e	Cundinamarca	1	(1.59)
	Subtotal		1	(1.59)
Total			49	(77.8)
1/2c,3c	1 /2c	Caldas	1	(1.59)
		Cundinamarca	1	(1.59)
		Norte de Santander	1	(1.59)
		Risaralda	1	(1.59)
	Subtotal		4	(6.35)
	3c	Cauca	1	(1.59)
		Boyacá	1	(1.59)
	Subtotal		2	(3.17)
Total			6	(9.52)
1/2a, 3a	1/2a	Boyacá	2	(3.17)
		Bogotá	1	(1-59)
	Subtotal		3	(4.76)
	3a	Risaralda	1	(1-59)
	Subtotal		1	(1.59)
Total			4	(6.35)
1/2b,3b	1/2b	Meta	1	(1.59)
		Bolívar	1	(1.59)
Total			2	(3.17)
4a,4c	4c	Nariño	2	(3.17)
	Subtotal		2	(3.17)
Total			2	(3.17)
Total			63	(100)

### Case definition

Our case definition is that of the U. S. Centers for Disease Control and Prevention (CDC) from 2019 ^20^.

### Phenotypical and serological analyses

Isolates obtained from Oxford and Palcam agar plates incubated at 37°C (± 2°C) for 22 hours (± 4 h), were phenotypically characterized by traditional biochemical methods and then by the semi-automated Vitek MS™ system (BioMérieux, Marcy l’Etoile, France). The control strain CLIP 4b used (Institut Pasteur reference strain) was kindly provided by Pasteur Institute to the *Instituto Nacional de Vigilancia de Alimentos* y *Medicamentos* (INVIMA). Isolates were serotyped as described by Seeliger and Höhne using commercial agglutination reagents (Denka Seiken, Tokyo, Japan) for somatic (O) and flagellar (H) antigens (https://bigsdb.pasteur.fr/_nuxt/img/serogroups.ff5cbf0.png). 

### Polimerase chain reaction

Serogrouping was conducted using multiplex polimerase chain reaction (PCR) as previously described ^8^. Interpretation of serogroup complexes identified by PCR corresponded to the following categories: (4b, 4d, 4e), (4a, 4c), (1/2a, 3a), (1/2b,3b,7), (1/2c, 3c).

### Antimicrobial testing

The minimal inhibitory concentration (MIC) for penicillin and trimethoprim/ sulfamethoxazole was determined according to the Clinical Laboratory Standar Institute (CLSI) 2016 guidelines for *L. monocytogenes*, guideline M45 ED3: 2016 with control strain Streptococcus pneumoniae 49619 ^21^.

### Molecular typing by pulsed field electrophoresis

Pulsed field electrophoresis (PFGE) analysis was performed following the PulseNet protocol with Apa\ enzyme ^15^. Briefly, the conditions used were the following: initial switch time of 4.0 s with final switch time of 40 s for 18 hours. Images were analyzed with Gelcompare 4.0. A cluster analysis on the fingerprints obtained by Apa\ was made with a similarity matrix calculation using the Dice coefficient followed by a dendrogram construction using the unweighted pair group method using averages (UPGMA) as algorithm with optimization and tolerance set to 1.5% with cluster grouping cut-off > 75% and patterns identity.

The regional comparison of the *L. monocytogenes* isolates from Colombia was made against the PulseNet Network Latin America and Caribbean maintained by the *Instituto Carlos Malbrán* in Argentina. A bundle file consisting of 42 isolates, representing Colombian isolates was utilized for this comparison.

### Whole genome sequencing

Of the 59 isolates characterized by PFGE Apa\ distribution groups (I and II), five isolates were selected for whole genome sequencing analysis. The genomic DNA was isolated by QIAamp DNA Mini Kit™ (Qiagen, Hilden, Germany)-lnvitrogen. Libraries were prepared for lllumina single pair and > 300 bp paired end sequencing using the lllumina Nextera XT™ Guide (15031942) (lllumina, Inc, San Diego, CA) and sequenced in a MiSeq™ sequencer (lllumina platform).

### Quality control, assembly and annotation

The fast quality control (FastQC) files were trimmed, to remove adapters and low-quality sequences using Trimmomatic (https://github.com/usadellab/Trimmomatic) subsequently, a quality analysis was carried out by FastQC (https://github.com/s-andrews/FastQC). De novo assemblies of the trimmed reads were constructed with SPAdes (https://github.com/ablab/spades). and the quality of the assemblies was evaluated using Quast (https://github.com/ablab/quast). The annotation of the genomes was performed with Prokka (https://github.com/tseemann/prokka).

### Multilocus sequence typing, serotyping and antimicrobial resistance

Multilocus sequence typing (MLST) was identified by the comparison of the assemblies on the Bacteria Isolate Genome Sequence Database (BIGSdb) for *L. monocytogenes* (BIGSdb-/m) (https://bigsdb.pasteur.fr/cgi-bin/bigsdb/bigsdb.pl?db=pubmlst_listeria_isolates). 

Serotyping of the isolates was determined by SRST2 (https://github.com/katholt/srst2) based on the trimmed reads. Detection of the genetic elements associated with antibiotic resistance were identified by the ABRIcate program (https://github.com/tseemann/abricate), using the following databases: NCBI, Comprehensive Antibiotic Resistance Database (CARD), ARG-ANNOT, Resfinder, MEGARes, PlasmidFinder and Virulence Factor Database (VFDB) and prophages with PFIAge Search Tool-Enhanced Release (PFIASTER) (https://github.com/arif-tanmoy/Using-PFIASTER).

### Phytogeny

Variant calling was made for each of the samples with Snippy and the EGD-e strain was used as the reference genome (genbank accession number: GCA_000196035.1), then a maximum likelihood phylogenetic tree was built with FastTree (https://github.com/morgannprice/fasttree) using SNP- sites https://github.com/sanger-pathogens/snp-sites).

### Regional Latin American phytogeny

A total of 54 SRA of *L. monocytogenes* regional public sequences retrieved from the Pasteur Institute repository were compared with Colombian sequences.

Raw sequence data were deposited at the NCBI and visualization with Microreact (https://microreact.org/).

## Results

### Epidemiological analysis of human isolates

The 63 *L. monocytogenes* strains characterized in this study were predominantly isolated from the city of Bogotá, and Antioquia, Valle del Cauca, Nariño, and Boyacá departments (n = 44; 69.8%). Most of these isolates were from females (n = 32; 50.7%), followed by males (n = 28; 44.4%), and a smaller proportion lacking gender data (n = 3; 4.7%). By age, the most frequent was the 15- to 54-year-old group (n = 21; 33.3%) followed by newborns (< 1 month; n = 14; 22.2%). Most of the isolates were recovered blood cultures (n =37; 58.7%) and cerebrospinal fluid (n = 12; 19.1%) (table 1). One isolate was associated with an outbreak in Cundinamarca in 2017; the other 62 isolates corresponded to sporadic cases.

### Phenotypical characterization - serological and PCR serogrouping

The 63 isolates identified as *L. monocytogenes* were characterized by PCR and corresponded to five serogroup-complex represented mainly by serogroup-complex 4b, 4d, 4e (77.8%) and serogroup 1/2c, 3c (9.5%) (table 2).

Nine serotypes were identified following the serogroup identification scheme (serological testing and PCR of each serogroup-complex): 4b,4e (46%), 4d,4e (30.2%), 1/2c (6.4%), 1/2a (4.8%), 4c (3.2%), 3c (3.2%), 4e (1.6%), 3a (1.6%), 1/2b (1.6%) (table 2). The three main serogroup-complexes 4b,4e, 4d,4e, 1/2c, represented 52 isolates (82.5%), mostly distributed in Antioquia, Bogotá, Boyacá, Nariño, Santander and Valle del Cauca. Serotype 4b,4e (29 isolates; 46%), was primarily detected in samples of different origins (table 2, supplementary table 1).

### Antimicrobial susceptibility

The 62 isolates evaluated were susceptible to penicillin and to trimethoprim/sulfamethoxazole.

### Genotyping by PFGE

**Figure 1.**
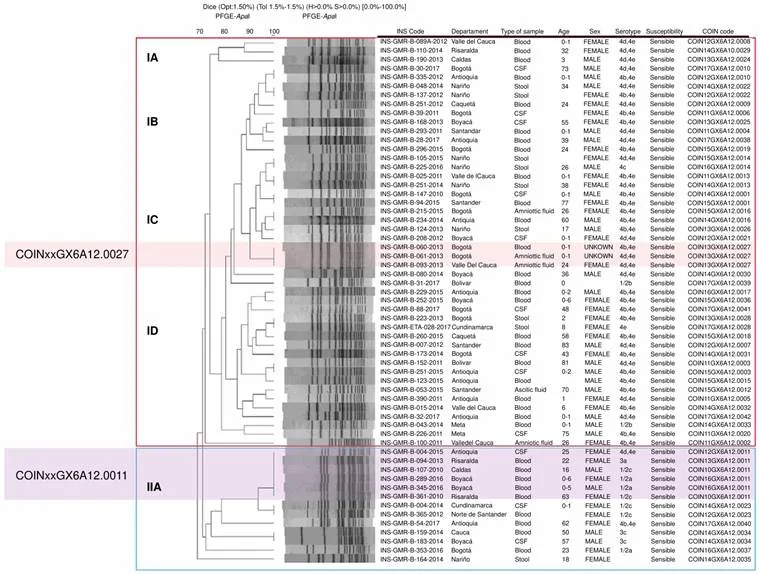
Dendrogram of genetic relationship of PFGE- ApaI patterns of 59 isolates of *Listeria monocytogenes* from Colombia (2010-2017). A total of 42 PFGE-*Apa*I patterns were associated with two principal groups (I-II). To the right of the figure frequent patterns are indicated: COINGX6A12.0027 (n = 3; 5.1%), COINGX6A12.0011 (n = 6; 10.2%), with a percent of similarity of 69.0%.

Using PFGE-Apa\, 59 isolates were categorized into two groups (I and II) with a similarity index of 69.0% (figure 1). The remaining 4 isolates were not typeable by PFGE.

PulseNet PFGE codes of *Listeria monocytogenes*: CO: Colombia; IN: *Instituto Nacional de Salud*; GX6: *Listeria monocytogenes*; A12: Enzyme ApaI.

Software GelCompare II, version 4.0, with a percent of tolerance and optimization of 1.5.

Group I included 46 isolates with 36 PFGE-*ApaI* patterns that showed at least 74% similarity. Isolates in this group I could be subdivided into IA to ID subgroups, distributed in 12 departments: Antioquia, Bolivar, Cundinamarca, Boyacá, Caldas, Caquetá, Meta, Nariño, Risaralda, Santander, and Valle del Cauca, and the city of Bogotá. The serotypes of group I were 4b, 4e, 4d, 4e, and 1/2b. This group included nine clusters (with 2 or more isolates) corresponding to patterns: COINGX6A12.0001, COINGX6A12.0003, COINGX6A12.0010, COINGX6A12.0013, COINGX6A12.0014, COINGX6A12.0016, and COINGX6A12.0028, without a specific relationship to serotypes or location. However, the pattern COINGX6A12.0027 was linked to serotype 4d, 4e and COINGX6A12.0022 to the Nariño department.

Group II included 13 isolates of six PFGE-*ApaI* patterns with a similarity of 69% (figure 1). This group was identified in Antioquia, Caldas, Boyacá, Cauca, Cundinamarca, Norte de Santander, and Risaralda and the city of Bogotá (table 3). Within this group, serotypes exhibited greater diversity, encompassing 4b,4e, 4d,4e, 1/2a, 1/2c, 3a, and 3c. This group had three clusters (with two or more isolates) sharing related with PFGE-Apal patterns: COINGX6A12.0011 without a specific serotype, and COINGX6A12.0023 and COINGX6A12.0034, which were related with serotypes 1/2c and 3c, respectively.

**Table 3.** Cluster grouping analysis by UPGMA PFGE-Apal of *Listeria monocytogenes* clinical isolates

Group	Similarity	Location	Subgroup	Similarity	Location	n	(%)
	UPGMA			UPGMA			
	(%)			(%)			
I	74	Antioquia	IA	77,6	Caldas	3	(5.1)
		Bogotá			Valle del Cauca		
		Bolívar			Risaralda		
		Boyacá Caldas	I B	88,9	Antioquia	10	(16.9)
		Caquetá			Bogotá		
		Cundinamarca			Boyacá		
		Meta			Caquetá		
		Nariño			Nariño		
		Risaralda Santander			Santander		
		Valle del Cauca	IC	82,6	Antioquia	15	(25.4)
					Bogotá		
					Bolívar		
					Boyacá		
					Nariño		
					Santander		
					Valle del Cauca		
			I D	72,1	Antioquia	18	(30.5)
					Bogotá		
					Bolívar		
					Boyacá		
					Caquetá		
					Cundinamarca		
					Meta		
					Santander		
					Valle del Cauca		
Subtotal						46	(78)
II	69	Antioquia				13	(22)
Subtotal		Bogotá					
		Boyacá					
		Caldas					
		Cauca					
		Cundinamarca					
		Nariño					
		Norte de Santander					
		Risaralda					
Total						59	(100)

### Regional comparison

**Figure 2.**
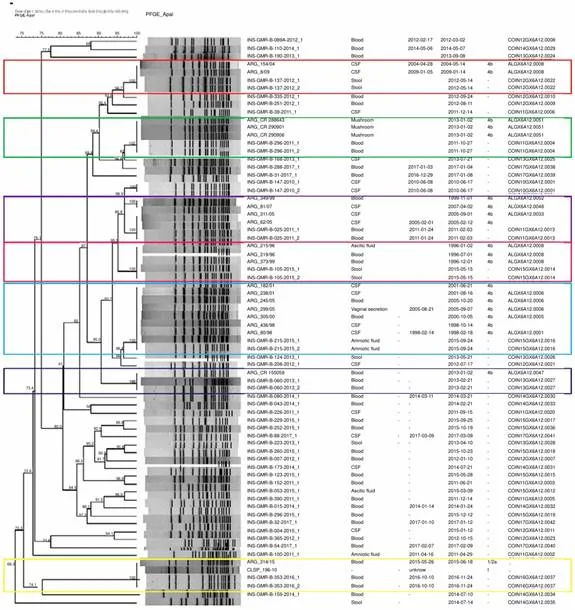
Regional comparison PFGE-Apal between clinical isolates of *Listeria monocytogenes* from Colombia and the regional database PulseNet Latin America and Caribbean Network. Right, are indicated the regional Latin America shared pattern with Argentina ALGX6A12.0033, ALGX6A12.0008, ALGX6A12.0006 related with Colombian national patterns COINxxALGX6A12.0013, COINxxALGX6A12.0014, COINxxALGX6A12.0016 respectively, and with Costa Rica ALGX6A12.0051, ALGX6A12.0047 related with Colombian national patterns COINxxALGX6A12.0004, COINxxALGX6A12.0027 respectively.

The clonal relationship of the strains identified in this study with those isolated in other Latin American countries was determined by PFGE-Apal. Five of the PFGE-Apal patterns observed in Colombian strains were also found in three other countries: Argentina, food (n =3 mushroom) and human (n = 5, blood; n = 7, cerebrospinal fluid; n = 1, ascitic fluid; n = 1 vaginal secretion); Costa Rica, human (n = 1 blood) and Chile with a single strain of unknown origin (figure 2).

PulseNet PFGE codes of *Listeria monocytogenes:* AL: America Latina; GX6: *Listeria monocytogenes*; A12: Enzyme Apa\.

Software GelCompare II, version 4.0, with a percent of tolerance and optimization of 1.5. Color box outlined, sharing pattern Argentina red, purple, fuchsia, blue, yellow; sharing pattern Costa Rica, green, dark blue.

### Whole genome sequencing

From four strains within group I identified by PFGE, single isolates were chosen for sequencing from each of the four subgroups (IA-ID), along with another isolate selected from group II (IIA) PFGE. The five genomic sequences corresponded to five distinct PFGE patterns, clustering into two groups via UPGMA, which were further integrated into the same phylogenetic cluster. These sequenced isolates differed in source, ranging from blood, cerebrospinal fluid, stool, and amniotic fluid. Additionally, the five isolates sequenced all belonged to serotype 4b based on *silico* analysis.

### MLST, ribosomal MLST rST, clonal complex CC and lineage

One sequenced isolate was identified as ST 1 with the other four isolates sequenced identified as ST2. rST identified were 15992 (2), 15983,15959, 98481. One sequenced isolate was identified as clonal complex 1 (CC1) and the other four sequenced isolates were identified as clonal complex 2 (CC2). And five isolates sequenced belonged to lineage I.

### Virulence genes analysis

The virulence factor database was used to evaluate the sequenced isolates for 40 virulence genes. All the sequenced isolates harbored the following genes related to virulence and metabolic functions

### Pathogenicity and virulence island

*LIPI-1 (Listeria Pathogenicity Island 1)*. The *prfA*-virulence gene cluster (pVGC) is the main pathogenicity island LIPI-1 (involvement in the intracellular infection cycle of *L. monocytogenes*) comprising (*prfA, plcA, hly, mpl,actA, plcB*); described as (*prfA*) \ described as (*prfA*) listeriolysin positive regulatory protein; (*plcA*) phosphatidylinositol-specific phospholipase c, exoenzyme; (*hlyA*) bacterial pore-forming toxin listeriolysin O precursor (LLO); (*mpl*); (*actA*) actin-assembly inducing protein precursor-biofilm formation; (picB) phospholipase C, exoenzyme, and (*iap/cwhA*) (invasion).

The internalins encoded within LIPI-1, which have different localizations, and the internalins (*inlA, inlB, inlF, inlJ, inlK*) related to invasion and adhesion interact with distinct host receptors to promote infections if human cells and/ or crossing of the intestinal, blood-brain, or placental barriers were present, alongside the action of genes such as (*bsh*), (*prsA2*), (*hpt*). 

*LIPI-3 (Listeria Pathogenicity Island 3)*. The pathogenicity island LIPI-3 (operon coding), LLS-bacteriocin and hemolytic cytotoxic factor integrate by the genes (*llsA*) listeriolysin S, (*llsB*), (*llsD*), (*llsG*), (llsH), *llsP*), (*llsX*) and (*llsY*). 

Also, by adhesion and invasion genes, (lpeA) lipoprotein promoting cell invasion and (*gtcA*), (*fbpA*), (*fss3*), (*lap*), (*lapB*), (*iap/cwhA*), (*vip*) and(*ecbA*); stress response (*clpCEP*); surface protein anchoring, (*lspA*); intracellular replication (lplA1), and immune response and modulation (*oatA*), (*pdgA*) and (*lntA*) (supplementary table 2).

### Antimicrobial resistance genes

Antimicrobial resistance genes resistance genes were identified in all *L. monocytogenes* sequences, *fosX* resistance to fosfomycin, *lin* resistance to lincosamides, *norB* resistance to quinolones, *mprF* confers resistance to cationic peptides that disrupt the cell membrane including defensins (supplementary table 2).

### Plasmid

The rep25_2_M640p00130(J1776 plasmid)_CP006612 was identified in all the sequences (supplementary table 2).

### Phages

Four phages were identified vB_LmoS_293_NC_028929(48) 43.48Kb (score >90) tail, virion, capsid, head, portal, terminase, integrase; LP_030_2_ NC_021539(30) 30.4Kb (score >90) terminase, protease, capsid,tail; LP_030_3_NC_024384(57) 41.2Kb (score >90) integrase, terminase, portal, capsid, tail; A118_NC_003216(4) 14.4Kb (score 70-90) lysin, tail (supplementary table 2).

### *Phytogeny global* Listeria

The phylogenetic tree included 54 sequences from 15 countries. The core genome SNP-based phylogeny defined 3 clusters from global comparison, the closest sequences of lineage I, serotype 4b, were in countries as France (SRR1100930), Chile (SRR3394973), Peru (SRR5972864) and India (SRR8383222). The previous Colombian sequences at Pasteur Database; (ERR1100922) serotype IIb, were grouped at the cluster of lineage I. Other sequences at the same lineage I, serotype 2b, were grouped with India, China, Nigeria, Francia, South Korea, Argentina and from serotype 4b with Venezuela. The other lineages II and III, were not related with the Colombia sequences (supplementary figure 1) (https://microreact.org/project/eKTACWSwERHWCdSZXGKxcx-listeria-monocytogenes). 

### Analysis matrix SNP

The analysis of matrix SNPs from some selected sequences of each lineage, identifies between 6 to 24452 SNPs. The shortest distance was with sequences of Chile (supplementary table 3).

### Publicly available International sequences of NCBI

*Data availability*. The five genome sequences of *L. monocytogenes* isolates were deposited at the National Centre for Biotechnology Information (NCBI) under the (Bioproject: PRJNA975580). And uploading to the *L. monocytogenes* (BIGSdb-lm) database. Last updated: 2023-07-26 (https://bigsdb.pasteur.fr/cgi-bin/bigsdb/bigsdb.pldb=pubmlst_listeria_isolates&page=query&prov_field1=f_country&prov_value1=Colombia&submit=1), as well as national data information (supplementary table 4). 

## Discussion

Our study examined 63 unique isolates of *L. monocytogenes* selected from a total of 446 Colombian isolates recovered in 2010-2017 from sporadic and outbreaks cases of listeriosis. These selected isolates underwent comprehensive characterization for both phenotype and genotype, with a subset of them undergoing further investigation through whole genome sequencing. This addition of WGS to our methodologies, recently implemented, serves to bolster the capabilities of the national laboratory surveillance, complementing established techniques like PFGE.

Due to the potential for severe outcomes and mortality associated with listeriosis, coupled with challenges in timely diagnosis, in some high income countries such as the United States and European nations, since the FDA and the Food Safety and Inspection Service ^22^ regulation have established that listeriosis is a disease requiring the mandatory reporting of cases and laboratory information about the pathogen. For instance, implementation of mandatory national reporting system in France (1999), allowed the complete capture of microbiological cases with improvements in the management and prediction of outcomes of listeriosis in a time window to prevent fetal losses (< 29 weeks of gestation and < 3 days of adequate management) ^23^. In contrast, in many Latin American countries like Brazil and Colombia, listeriosis is not a mandatory reportable disease. As a result there is limited data about its occurrence and outcomes in these countries, except it has become mandatory for countries as Chile, since 2004 ^24^, Uruguay from 2012 ^25^ and Argentina in 2022 ^26^.

It is crucial to emphasize the significance of identifying and surveilling serotypes and PFGE patterns in cases and outbreaks, particularly in relation to food types. Currently, in Colombia many reports of sporadic cases with fatal and severe consequences lack complete characterization. This highlights the urgent need for comprehensive identification and surveillance protocols to effectively address listeriosis within the country.

In 2005, a meningitis case caused by *L. monocytogenes* was identified in a 14-year-old patient in Bogotá. However, the specific serotype implicated in the infection was not defined ^27^. In the outbreak report from 2009 fresh cheese was possibly related with serotypes 4b, 4d-4e and 1/2a from food sampling analysis without clinical serotype confirmation ^28^. In 2011, an unusual case was reported in Antioquia involving brainstem encephalitis and myelitis, a combination rarely seen. This severe condition, leading to fatal consequences, affected an immunocompetent patient and was linked to the consumption of unpasteurized dairy products contaminated with *L. monocytogenes*. However, no specific serotype was reported in association with the case ^29^. For this study, an outbreak of 2017, in Cundinamarca, with 11 persons affected, was related with serotype 4e and PFGE-*Apa*I pattern COIN17GX6A12.0028, this serotype 4e also have been reported in food types ^30^.

There are few reports of maternal-neonatal listeriosis in Colombia. In 2015, a meningitis case in a neonate sensitive to ampicillin with fatal outcome was reported from Bogotá ^31^. We identified 5 (7.94%) related cases of perinatal listeriosis and 14 (22.2%) associated with newborn cases.

That evidence of invasive disease resulting in fatal outcomes is substantiated by a review of the records from the *Sistema Nacional de Vigilancia en Salud Pública* (SIVIGILA) and the *Registro Único de Afiliados* (RUAF). These records document cases of perinatal and late neonatal mortality associated with *L. monocytogenes*. During the study period, twenty-two cases of pregnancy-related illnesses associated with listeriosis were reviewed, along with related cases in newborns and the corresponding samples of amniotic fluid ^4^, cerebrospinal fluid ^4^, and blood cultures ^14^. One death from Boyacá was attributed to *L. monocytogenes*. This case was confirmed by laboratory testing (blood culture) in a female newborn of 6 days, with serotype 1/2a. In the other twenty-one cases, the causes are not specified or related.

In clinical isolates of *L. monocytogenes* multidrug resistance remains infrequent with only a few cases reported ^32^. Based on national reports, a study conducted in 1999 in a clinical setting in Valle del Cauca, which included 19 cases of meningitis in immunosuppressed patients-possibly associated with consumption of raw food- identified sensible isolates to ampicillin and penicillin in 97.3%, with a decreased susceptibility to trimethoprim/sulfamethoxazole in 17.1% ^33^. Despite these clinical reports, the national surveillance conducted by *Grupo de Microbiología* at the *Instituto Nacional de Salud* on clinical isolates received has remained sensible during these years (from 2010-2017).

PFGE-*Apa*I typing unveiled a notable diversity among *L. monocytogenes* isolates, with a genetic similarity reaching 69%. This level of similarity aligns with findings from other studies ^34^. This variability is represented by small clusters and can be attributed to high genetic recombination and differences in the content of mobile element genes ^35^^-^^37^.

As have been reported by other researchers, different serovars can be grouped in the same PFGE patterns ^38^^,^^39^ supported in research into the evolutionary analysis of lineage I ^9^. This indicates that serotype 4b evolved once from originated from serotype 1/2b-the likely ancestral serotype of lineage I-while lineage II serotype 1/2c has serotype 1/2a as its ancestor.

In our study, the dendrogram analysis found this association with lineage I between serotypes 4b and 1/2b; For lineage II, the serotypes 1/2c and 1/2a are present at the same group ^9^^,^^40^.

Twelve clusters with more than two isolates and with identical PFGE pattern related were identified. Of these, only three were present at the same year and but at different locations, suggesting a common source of contamination. Additionally, through the years one main cluster was related with persistence in different departments, a characteristic described for *L. monocytogenes* due to few genetic variations, which leads to infections that persist for several years ^37^.

Using the regional surveillance of *L. monocytogenes* by PulseNet Latin America and Caribbean Network, 42 PFGE patterns representative of Colombia enables us to discern the circulation of isolates within Latin America. Our analysis revealed that three patterns were shared between Colombia and Argentina, and two with Costa Rica, underscoring their significance in dissemination. In September, 2025, through PulseNet Latin America and Caribbean Network, as response to a requirement from Argentina -which was established a nexus between human cases of listeriosis and a common source verified by genomic analysis- after a regional comparison, no relation was established, with the conglomerate of cases from Argentina with a difference of more than 100 SNPs with México (150-245 SNPs) and Colombia (118-123 SNPs) (PulseNet Latin America and Caribbean Network, data not published).

Previous reports found that human listeriosis clusters can be widely distributed geographically across countries and continents with an increase of such clusters over the last decade (2008-2018) of reporting. For example an estimated 17% of cluster events involved two or more countries ^41^, as found in a report with a strain confirmed by PCR as group 4b with MLST ST6 and a PFGE profile indistinguishable across European countries ^42^.

In another report from 2015 by PulseNet USA Network, a cluster was identified that was highly related by PFGE and wgMLST in clinical isolates of *L. monocytogenes* from packaged salad affected nine states in USA and Canada. There was evidence of the link between contaminated food and human illness ^43^ and involved different countries as 2018 with cantaloupe melons from Australia were distributed to eight countries ^44^ which reveal as have enhanced surveillance with advance technologies as WGS and regulations.

The genomics analysis of *L. monocytogenes* from human cases confirmed initial findings regarding potential virulence factors, pathogenicity island, and their phylogenetic relationship within these pathogens in Colombia. Thus, it was possible to establish the circulation of lineage I, ST1, ST2, CC1, CC2 which are closely linked to human disease and predominantly belong to the serotype 4b ^45^^,^^46^. These strains are regarded as epidemic clones, exhibiting potential hypervirulence characterized by enhanced efficiency in gut colonization and fecal shedding. Moreover, they are implicated in neuro-invasiveness, particularly strains from (CC1, CC2, CC4 and CC6) ^10^^,^^47^. In these sequences, it was identified PrfA, the master regulator directly involved in the control and expression of virulence genes ^48^.

Between sequences analyzed, factors contributing to increased colonization and hypervirulence were identified, including full length *InIA,* LIPI-1, and LIPI-3.

To indicate invasive illness, the presence of chromosomal island 1 (LIPI-1), is necessary, as identified in these sequences. LIPI-1 is composed of genes essential for intracellular growth, host invasion, and cellular proliferation ^49^. Other virulence clusters present at those *L. monocytogenes* sequences, contains an operon of two genes (*inlA/B*) of internalins necessary for the attachment to and invasion of non-phagocytic host cell ^48^^,^^50^. LIPI-3, described in lineage I, and CC1 and CC4, encodes cluster hemolysin, listeriolysin S (LLS), a virulence factor with hemolytic and cytotoxic activity that contributes to the spontaneous and epidemic outbreaks ^51^^,^^52^. The strains with truncated and nonfunctional virulence factor *InIA* protein exhibit reduced pathogenicity and loss of virulence ^53^. At these sequenced isolates from Colombia, the protein *InIA* was complete, with an identity of 96.75-96.71% and a coverage of 100%, and with *InIB* protein are the major virulence factor involved in cell invasion as a cause systemic human infection ^19^.

*In silico* analysis revealed the presence of intrinsic resistance genes associated with third generation cephalosporins, fosfomycin, lincosamides, quinolones and resistance to cationic peptides ^54^^,^^55^. Those traits of genes *fosX, lin, mprF, norB* and *mgrA* have been identified in clinical samples, between most common genes found in six world regions *fosX* and *lin* genes, followed by *abc-f, tet(M)*, and *vanRST, vanXY-C* in lower frequency and were not found no *ampR* and *aac(6”)* and *aph(2”)* genes with medical relevance. It is expected to find more diversity of antimicrobial resistance genes in the environment, with possibility to be transferred to humans ^56^.

Between the mobile genome elements, in each sequence three prophages were identified intact (score > 90) vB_LmoS_293_NC_028929; P_030_3_ NC_024384; LP_030_2_NC_021539 and one with a A118_NC_003216 (score 70-90) and one plasmid J1776. The phages are related with virulence and pathogenicity of global distribution associated with the survival evolution and persistence in food processing facilities ^57^.

Plasmids in *L. monocytogenes* carry a variety of genes beneficial for their host associated under particular conditions, such as stress response, disinfectants and antibiotics, with survival advantages ^58^.

Studies indicate that prophages vB_LmoS_293, A118_NC_003216, P_030_3_NC_024384 and plasmid J1776 were identified in sequences present in human cases ^59^. In other studies J1776 plasmid belonged to different clonal complex and type sequences CC6, ST6 and CT434 with *cadA* gene relation in food production, animals and environment ^60^.

Phylogenetic comparison with isolates from other countries demonstrated sequences closely related to lineage I, which are globally distributed and related with serotypes 4b and 1/2b with regional countries like Perú, Chile, Venezuela. The SNP distance was between countries of lineage I (29 SNPs); in contrast, with Chile and the others there was more distance. The relationship with isolates from Chile, would suggest dispersion and circulation of the strains in the region. The representation of *L. monocytogenes* Colombia sequences human origin in global platforms as Pasteur the BIGSdb-Lm database (https://bigsdb.pasteur.fr/listeria), facilitates the integration of genomic surveillance, to improve the public health response in the country.

In this study, the analysis of *L. monocytogenes* demonstrates the genetic diversity by different methodologies in a difference of sensitive and high specific method as genomics was defined for some lineages, clonal complex, and confirmed serotypes, virulence and resistance genes as well as mobile genetic elements associated with potential of outbreaks and disease severity as identified a public health problem in population.

Listeriosis constitutes a serious problem of public health in Colombia. Intensified surveillance efforts, including genomic analysis, are Imperative to implement timely control and prevention measures. The morbidity and mortality associated with this pathogen underscore the urgency of such interventions.

Our laboratory-based surveillance of *L. monocytogenes* leads us to present four conclusions in the context of Colombia and the region:

1. The notification and reporting of outbreaks and sporadic cases of *L. monocytogenes* should be made mandatory for Colombia.
2. Improving the notification and monitoring of foodborne disease caused by *L. monocytogenes* is essential to prevent the spread of the pathogen and to establish correlations between affected population and the implicated food sources.
3. Pregnant women are a high-risk group for listeriosis due to the serious consequences this disease can have on the fetus and newborn. Therefore, they should focus on prevention strategies.
4. It is necessary to strengthen laboratory-based surveillance with inter institutional collaboration during suspected outbreaks to help recognize the source of infections and improve control measures. Such surveillance should include the reporting of serotypes and identification of persistent specific clones, lineages, virulence, antimicrobial resistance genes and genome accessories from human and food sources.
